# Identification of novel compound heterozygous *SPG7* mutations-related hereditary spastic paraplegia in a Chinese family: a case report

**DOI:** 10.1186/s12883-018-1199-9

**Published:** 2018-11-29

**Authors:** Xiaoqian Zhang, Lei Zhang, Yanqing Wu, Gang Li, Shengcai Chen, Yuanpeng Xia, Hongge Li

**Affiliations:** Department of Neurology, Union Hospital, Tongji Medical College, Huazhong University of Science and Technology, No.1277, Jiefang avenue, Wuhan, 430022 Hubei China

**Keywords:** Hereditary spastic paraplegias, Compound heterozygous *SPG7* mutations, Genetic diagnosis, Next generation sequencing, Paraplegin

## Abstract

**Background:**

Autosomal recessive hereditary spastic paraplegias (ARHSPs) are a group of clinically and genetically heterogeneous neurodegenerative diseases with progressive spasticity and weakness in the lower limbs. Mutations in the *Spastic Paraplegia gene 7 (SPG7)* account for about 5–21% of ARHSP cases. However, in Asians, few reports about the mutations exist. In this study, we firstly report a novel finding from a Chinese family with compound heterozygous *SPG7* mutations, in which three siblings were affected with a complicated form of ARHSP.

**Case presentation:**

A 56-year-old man presented with progressive stiffness, weakness and ataxia in the lower limbs. Two sisters of him had similar symptoms and dysarthria. Brain magnetic resonance imaging (MRI) revealed cerebellar atrophy in each of the patients. Genetic analysis, which exerted a targeted next generation sequencing (NGS) panel covering 917 comprehensive ataxia genes to the proband, followed by Sanger sequencing of candidate genes in other eight family members, was used to find the etiology of the disease. Ultimately, we identified compound heterozygous *SPG7* mutations with two mutations: (c.1150_1150-1insCTAC and c.2062C > T, p.Arg688Trp) and one single nucleotide polymorphism (c.2063G > A, p.Arg688Gln).

**Conclusions:**

The four bases insertion mutation (4bIM) was predicted to cause frameshift mutation or affect the splicing, and the last two variants were led to a stop codon mutation (p.Arg688Ter). As located in highly conserved positions and encoded paraplegin, the mutations were speculated to result in a truncated or defective protein and would be pathogenic factors of the disease. This paper proves to be the first case report of *SPG7* mutation in ARHSP reported in Chinese population. Our findings widen the spectrum of *SPG7* mutations of ARHSP and indicate that the *SPG7* mutation is an important cause of adult-onset undiagnosed ataxia.

**Electronic supplementary material:**

The online version of this article (10.1186/s12883-018-1199-9) contains supplementary material, which is available to authorized users.

## Background

Hereditary spastic paraplegias (HSPs), characterized by progressive spasticity and weakness in the lower limbs, are a clinically and genetically heterogeneous group of neurodegenerative disorders due to retrograde axonal degeneration of the corticospinal tracts [1, 2]. Clinically, HSPs could be divided into pure and complicated forms according to the absence or presence of neurological and extraneurological features, such as ataxia, cognitive impairment, optic atrophy, dementia, peripheral neuropathy, extrapyramidal features, amyotrophy, sensory neuropathy and epilepsy [2, 3].The genetic basis of HSPs are complex and more than 76 monogenic subtypes involving in all modes of inheritance (autosomal dominant, autosomal recessive, X-linked, and mitochondrial) have been described [4, 5]. As the large genetic and phenotypic overlapping among HSPs, making a correct diagnosis is difficult.

Recent advances in next generation sequencing (NGS) technology makes it more accurate and faster to diagnose the highly heterogeneous diseases [6]. Here, we first described the clinical characteristics of a Chinese family with three individuals affected with a complicated form of ARHSP. Then we used targeted next generation sequencing in the index case and verified candidate genes on other family members and finally identified compound heterozygous mutations in the *SPG7* gene.

Spastic paraplegia 7 (SPG7, OMIM#602783), as one of the most common form of autosomal recessive hereditary spastic paraplegia (ARHSP) caused by mutations in the *SPG7* gene, was firstly identified in 1998 [7]. The *SPG7* gene is located in chromosome 16q24.3 and comprises 17 exons spanning approximately 52 kilobases [8]. Paraplegin, a 795-amino acid protein encoded by *SPG7*, is a mitochondrial metalloprotease localized at the inner mitochondrial membrane and belongs to the AAA protein superfamily which play a significant role in different cellular activities [7, 9]. In this study, we proposed the mutations in *SPG7* were resulted in a truncated or defective protein and would be pathogenic factors of the disease.

## Case presentation

**Fig. 1:**
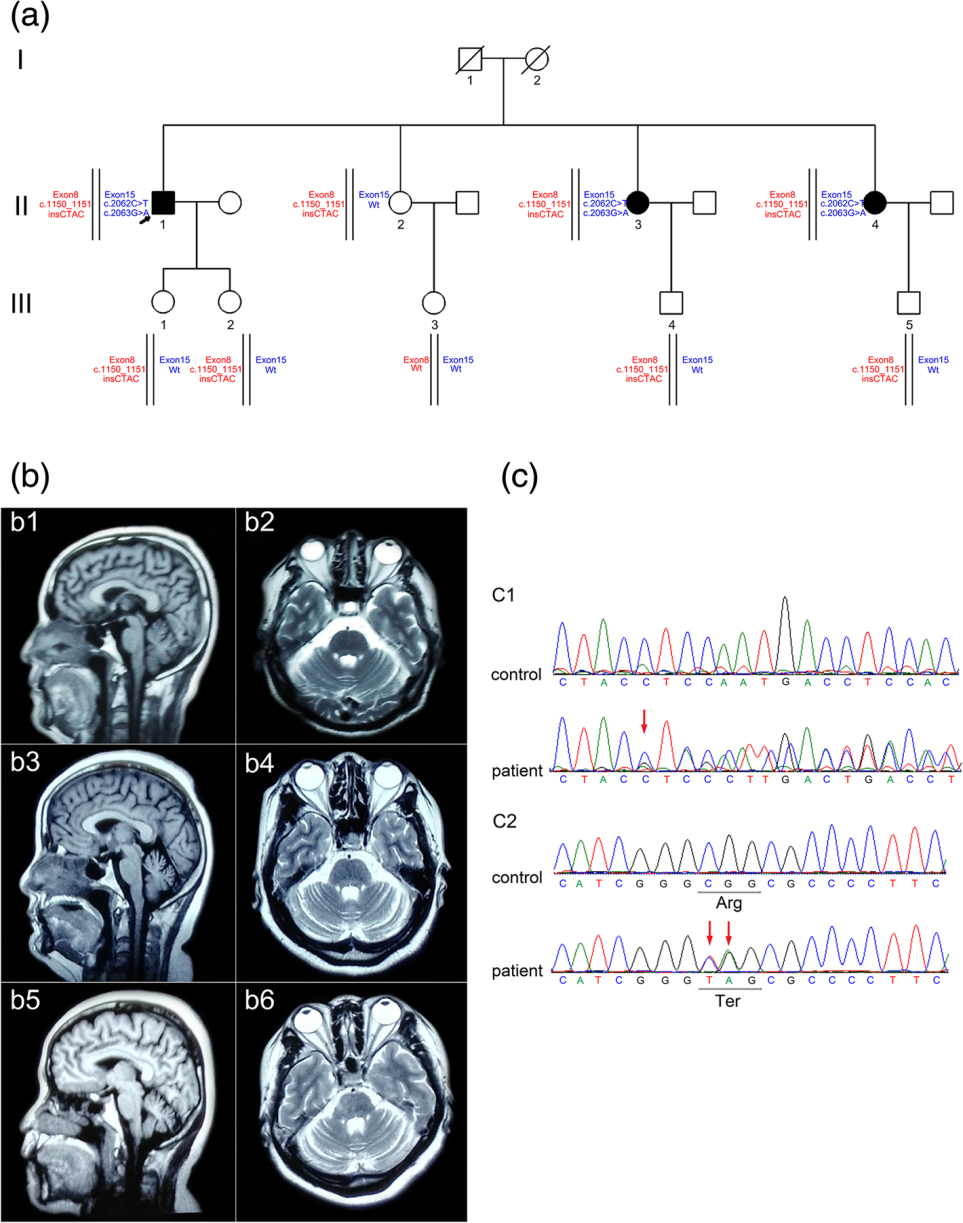
Pedigree, brain MRI and mutation sites of the patients. **a** Pedigree and the *SPG7* mutations of the Chinese family. The proband (II-1) and another two siblings (II-3 and II-4) are compound heterozygous for both variants (c.1150_1150-1insCTAC and p.Arg688Ter) and were affected with HSP. The other immediate family members are either heterozygous for only one sequence variant (c.1150_1150-1insCTAC) or wild type, and all are asymptomatic. **b** Brain MRIs of the patients in 2016. Sagittal T1-weighted (b1, b3, b5) and transverse T2-weighted (b2, b4, b6) images showed mild cerebellar atrophy in II-1 (b1, b2) at age 54, in II-3 (b3, b4) at age 48 and in II-4 (b5, b6) at age 44. The corpus callosums of them were normal. **c** Electropherograms of a control with the *SPG7* wild-type sequence (upper) and a patient with heterozygous mutations (lower). The mutation sites were indicated by red arrows. **c1** Four bases insertion heterozygous mutation (c.1150_1150-1insCTAC). **c2** Two adjacent sites single-base substitution variations in exon 15 resulted in a stop codon mutation (p.Arg688Ter)

A family from a small country in the middle of China was carefully analyzed (Fig. 1a). The index case is a 56 years old male, his parents were already dead (at the age of 60) and had no neurological deficits according to his recall. There is no consanguinity in this family. After obtained written informed consent, all participants underwent standard neurological examination by the same experienced neurologist, and three individuals were found to be suffered with stiffness, pyramidal weakness, extensor plantar response, ataxia or dysarthria (Table 1).

**Table 1:** Clinical features and neurophysiologic findings of the three affected siblings

Patient characteristics	II-1	II-3	II-4
Gender	Male	Female	Female
Age at onset (years)	45	39	34
Age at examination (years)	55	49	45
Past history	Hyperlipidemia	Hypertension Lumbar disc herniation	Hypertension Brain stem infarction
Motor disability	Unable to walk without aids	Able to walk with aids	Wheelchair-bound
Gait	Ataxia	Ataxia	Spastic-ataxia
Upper limb			
Spasticity	–	–	+(Left)
Weakness	–	–	+(Left)
Ataxia	–	–	–
Hyperreflexia	–	–	+(Left)
Sensory impairment	–	–	–
Lower limb			
Spasticity	–	–	+(Left)
Weakness	–	–	+(Left)
Ataxia	++	+	+
Hyperreflexia	++(Bilateral)	+(Bilateral)	++(Left)
Sensory impairment	–	–	–
Plantar reflexes	++(Bilateral)	+(Bilateral)	++(Left)
Dysarthria	–	+	+
Nystagmus	–	–	–
Bladder dysfunction	–	–	–
Cognitive function	–	–	–
Auxiliary examination			
Brain MRI	Cerebellar atrophy Scattered ischemic foci in left temporal lobe	Cerebellar atrophy Scattered lacunar infarcts in the right basal ganglia	Cerebellar atrophy Brainstem old cerebral infarction
EMG/NCS	Normal	Note done	Note done
EEG	Note done	Note done	Note done

– = absent; + = mild (or active); ++ = moderate (or hyperfunction); NCS = nerve conduction studies

### Clinical history

Patient II-1 (the proband) was initially presented at our outpatient clinic with progressive difficulties with gait and balance. From 11 years ago, he gradually felt stiffness and weakness of his lower limbs. In last two years, the symptoms were obvious, in addition with gait disturbance and intermittent numbness. Physical examination showed symmetric spasticity of the legs with hyperreflexia, bilateral Babinski sign (+) and scissors gait. Brain MRI in 2016 revealed mild cerebellar atrophy (Fig. 1b, b1 and b2).

Patient II-3 was 50 years old and was his elder sister. From 11 years ago, she had suffered the same symptoms like the proband. She also had waist pain, numbness in her left hand and mild dysarthria. Neurologic examination revealed dysarthria, active tendon reflexes in the lower limbs and positive Romberg’s sign. Brain MRI revealed mild cerebellar atrophy (Fig. 1b, b3 and b4).

Patient II-4 was 46 years old and was his youngest sister. At age of 34 years, she complained of difficulty in walking and weakness of bilateral lower limbs, which lead her to tumbled easily. She also suffered from dysarthria. After an ischemic stroke, the symptoms were getting worse. Physical examination showed ataxia, hypermyotonia, tendon hyperreflexia and muscle weakness in the left body. Brain MRI showed mild cerebellar atrophy and old brainstem cerebral infarction (Fig. 1b, b5 and b6).

The last sibling of the proband was a 53 years old woman (II-2). Like all the younger generation (III-1, III-2, III-3, III-4, III-5), she had not complaint about any neurologic symptoms.

### Genetic analysis

Blood samples were obtained from the whole family. Genomic DNA was extracted from EDTA-anticoagulated peripheral blood by using standard methods. As all the three affected individuals manifested ataxia and cerebellar atrophy, we first detected spinocerebellar ataxia genes (*SCA1, 2, 3, 6, 7, 12* and *17*) by using PCR fragment analysis in the proband. The results were negative (Additional file 1: Table S1, Additional file 2: Figure S1). To further examine other genetic mutations, the targeted next generation sequencing involving 917 comprehensive ataxia genes panel (Additional file 3: Table S2) was performed. Using genomic DNA, all exons, untranslated regions (UTRs) and the variable splice sites of the relevant genes were sequenced and aligned to reference sequences on the human genome by using standard methods. Then the screened variants were filtered against dbSNP142 (ftp://ftp.ncbi.nih.gov/snp/organisms/human_9606), Human reference genome (ftp://hgdownload.cse.ucsc.edu/goldenPath/hg19), the 1000 Genomes project (ftp://ftp-trace.ncbi.nih.gov/1000genomes), OMIM database (http://omim.org/) and so on. Prediction of pathogenicity was assessed by the PolyPhen 2, SIFT, MutationTaster or FATHMM software and so on. All of them are calculate tools to predict the possible impact of the variants on the structure and function of proteins [10, 11]. Classification of sequence variants was based on the American College of Medical genetics and Genomics (ACMG) recommendations for interpretation and reporting of sequence variations [12]. Finally, for economic reasons, another two affected siblings, one healthy sibling, and the healthy offspring were only targeted detected and confirmed candidate genes by conventional Sanger sequencing.

Subsequent targeted NGS panel covering 917 relevant genes finally identified three heterozygous variations (c.1150_1150-1insCTAC, c.2062C > T, c.2063G > A) in *SPG7* gene in this family. These variations were confirmed by Sanger sequencing (Fig. 1c). 99.1% of the target regions were included in the NGS, with an average depth of 153.46x and 97.93% of the target regions were covered by at least 20 reads.

The first was a four bases insertion heterozygous mutation (4bIM) between chr16:89598474 and chr16:89598475 of the *SPG7* gene. Besides, two heterozygous missense variants (c.2062C > T, p.Arg688Trp and c.2063G > A, p.Arg688Gln) in exon 15 of the *SPG7* gene were also validated. The phylogenetic validation of the above gene mutations showed that the two affected sisters also had the same variations. The majority of asymptomatic individuals in the family, including the sibling (II-2) and offspring (III-1, III-2, III-4, III-5), only carry a 4bIM in heterozygous state (Fig. 1a, Additional file 4: Table S3). In addition, in the index case, three other missense mutations including c.316C > T, (p.Arg106Cys), c.71G > A (p.Arg24His) and c.952A > G, (p.Thr318Ala) were also found in *AFG3L2* exon 4, *PPP2R2B* exon1, and *SLC1A3* exon 5, respectively. More additional detailed variations and annotations found in the proband can refer to Additional file 5: Table S4.

## Discussion and conclusions

In this report, we described the clinical features and genetic analyses of a non-consanguineous Chinese family including three affected siblings with a complicated form of ARHSP. Using NGS, we finally mapped the disease loci to two mutations (c.1150_1150-1insCTAC and c.2062C > T, p.Arg688Trp) and one single nucleotide polymorphism (c.2063G > A, p.Arg688Gln) in the coding sequences of the *SPG7* gene.

Until now, at least 77 different mutations of the *SPG7* gene including missense, nonsense, splicing, frameshift and exonic deletions have been described in the literatures [13–19]. Although SPG7 accounts for 5 to 21% of ARHSPs, with a prevalence estimated at 2–6/100,000 [20–22], it has never been reported in a Chinese population. Our findings suggested that spastic paraplegia 7 might be neglected as an etiology in China.

Mutations in *SPG7* are known to present both pure and complicated phenotypes. In the complicated phenotypes, the clinical symptoms could be accompanied by cerebellar ataxia, optic neuropathy, and cognitive impairments [23–25]. Cerebellar atrophy detected by brain MRI is the most common feature in some subjects [18, 19]. Consistent with their reports, cerebellar atrophy with dysarthria and ataxia are also the major characteristics of our patients. The average age at onset of the three patients is 39 years (range 34–45 years), similar to previous majority described SPG7-HSP families, in which age at onset differed from 10 to 45 years [4, 18].

The first identified sequence variation is a four bases insertion mutation (c.1150_1150-1insCTAC, 4bIM) located between chr16:89598474 and chr16:89598475 of the *SPG7* gene (just in the interval of exon 8 boundary and the GT splice donor site). According to the ACMG criterion, it was regarded as an uncertain significance mutation. However, because of the limitations of the next generation sequencing technology, we only sequenced at the DNA level and did not further analyze it at the cDNA level, so we could not identify the exact impact of this mutation. We suspect that it may cause frameshift mutation or affect the splicing. Another identified sequence variation c.2063G > A, p.Arg688Gln in exon 15 is a known polymorphism [18], with a variation frequency of 10.58% in the 1000g2015aug_ALL. But, co-mutation with c.2062C > T, p.Arg688Trp (adjacent site, proved to be on the same haplotype) does generated a novel stop codon mutation at the 688th amino acid (p.Arg688Ter) in our study. The two missense variants had an in cis configuration, and the segregation analysis supported the variations of the c.1150_1150-1insCTAC and p.Arg688Ter of the three patients were derived from their father and mother, respectively. Unfortunately, we were unable to verify the sources of variations in this family, as the blood samples of parents ((I-1, I-2) were not available.

To our knowledge, the c.1150_1150-1insCTAC mutation and p.Arg688Ter mutation identified in this study are firstly reported and both located in the highly conserved domain of paraplegin (AAA-domain; Peptidase M41 domain) [26]. They were predicted to result in a truncated or defective protein involved in the degeneration of corticospinal tract and cerebellar Purkinje neurons, which could result in progressive spasticity, weakness in the lower limbs and ataxia. Previous studies found that, in paraplegin-deficient mice, there were morphologically abnormal mitochondria in distal regions of affected axons and damage of anterograde axonal transport [27]. Therefore, our finding suggested a pathogenic role of the four bases insertion mutation and of the stop-codon mutation in *SPG7*.

In the proband, we also revealed another heterozygous missense mutation c.316C > T, p.(Arg106Cys) in exon 4 of *AFG3L2*, which resulted in an amino acid change from arginine to cysteine at codon 106. According to the ACMG criterion, it was regarded as an uncertain significance mutation. Mutations in *AFG3L2* have been reported to cause dominant ataxia SCA28 [28]. The AFG3L2 is a mitochondrial protein coded by *AFG3L2* gene and is the binding partner of paraplegin on the inner mitochondrial membrane. Both of them are abundant in cerebellar Purkinje neurons, deep cerebellar nuclei, neocortical pyramidal neurons, and motor neurons in the brain stem and participated in mitochondrion ribosomal assembly, proteome quality control, and mitochondrial DNA maintenance [28–30], so it is not surprising that they can present the same symptoms such as stiffness, pyramidal weakness, extensor plantar response and ataxia. Herein, we speculated that the mutation might be unrelated to the disease in the family, for it exists in a healthy family member (III-1) but absents in another affected family member (II-4). Likewise, another two missense mutations c.71G > A, (p.Arg24His) in *PPP2R2B* exon1 and c.952A > G, (p.Thr318Ala) in *SLC1A3* exon 5 were also excluded because they do not exist in all affected family members or exist in healthy family members*.*

In conclusion, our study successfully reaches a final molecular diagnosis in this family by using next generation sequencing technology and broadens the spectrum of the mutations on *SPG7*. Next generation sequencing maybe a cost-effective and practical approach to diagnose rare or highly heterogeneous neurodegenerative disorders, especially when genotype-phenotype correlations are still unclear at present. Besides, our results suggest that SPG7 should be considered in undiagnosed ataxia in the future.

## Additional files

Additional file 1: **Table S1.** Dynamic mutation test results. It shows the triple nucleotide (CAG) repeat numbers of the spinocerebellar ataxia-related genes (*SCA1*, *SCA2, SCA3, SCA6, SCA7, SCA12* and *SCA17*) in the proband. (DOCX 22 kb)

Additional file 2: **Figure S1.** Electrophoregram of the proband. It is an electrophoregram of the numbers of CAG repeats for the genes associated with spinocerebellar ataxia in the proband and healthy controls. (DOCX 53 kb)

Additional file 3: **Table S2.** The ataxia gene panel. It is a list of hereditary ataxia-related genes analysed in the proband. (XLSX 16 kb)

Additional file 4: **Table S3.** Family verification results. It is the first generation verification results of the variants mentioned in the manuscript in this family including three patients and the asymptomatic subjects. (DOCX 23 kb)

Additional file 5: **Table S4.** Variation annotations of the proband. It is an excel table containing the detailed annotations of all the variations validated in the proband. (XLSX 4239 kb)

## Abbreviations

ACMG: American College of Medical Genetics and Genomics
ARHSP: Autosomal recessive hereditary spastic paraplegia
MRI: Magnetic resonance imaging
NGS: Next generation sequencing
SCA: Spinocerebellar ataxia
SNP: Single nucleotide polymorphism
SPG7: Spastic paraplegia 7
