# Supplementary material for: Identification of novel compound heterozygous SPG7 mutations-related hereditary spastic paraplegia in a Chinese family: a case report
Source: BMC Neurol. 2018 Nov 29;18:196. doi: 10.1186/s12883-018-1199-9 (PMC6263041; doi:10.1186/s12883-018-1199-9)
Supplement: Supplementary file 1 — Table S1. Dynamic mutation test results. It shows the triple nucleotide (CAG) repeat numbers of the spinocerebellar ataxia-related genes (SCA1, SCA2, SCA3, SCA6, SCA7, SCA12 and SCA17) in the proband. (DOCX 22 kb) [file 12883_2018_1199_MOESM1_ESM.docx]

**Table S1** Dynamic mutation results of spinocerebellar ataxia-related genes in the proband.

| Disease | Gene | Position in the electropherogram | Repetition in Normal population | Reference range（**bp**） | Result |
| --- | --- | --- | --- | --- | --- |
| SCA1 | *ATXN1* | 1 | <39 | <242 | normal |
| SCA2 | *ATXN2* | 2 | <32 or >79 | <187 | normal |
| SCA3 | *ATXN3* | 3 | <45 or >86 | <310 | normal |
| SCA6 | *CACNA1A* | 6 | <19 or >33 | <159 | normal |
| SCA7 | *ATXN7* | 7 | <38 or >130 | <373 | normal |
| SCA12 | *PPP2R2B* | 12 | <51 or >78 | <257 | normal |
| SCA17 | *TBP* | 17 | <45 or >63 | <263 | normal |
