# Supplementary material for: Identification of novel compound heterozygous SPG7 mutations-related hereditary spastic paraplegia in a Chinese family: a case report
Source: BMC Neurol. 2018 Nov 29;18:196. doi: 10.1186/s12883-018-1199-9 (PMC6263041; doi:10.1186/s12883-018-1199-9)

**Figure S1**, Electrophoregram of dynamic mutation detection of spinocerebellar ataxia in the proband. Lane N and lane SN represent the results of CAG repeat number detection of the proband and the healthy control, respectively.


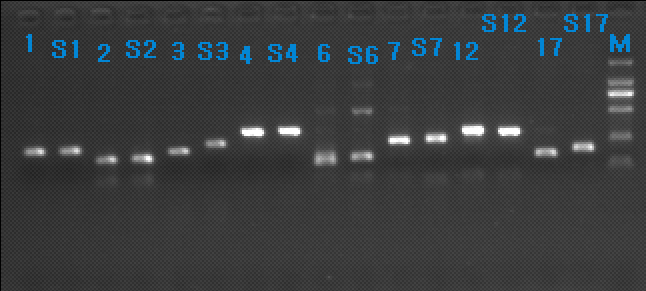

Supplement: Supplementary file 2 — Figure S1. Electrophoregram of the proband. It is an electrophoregram of the numbers of CAG repeats for the genes associated with spinocerebellar ataxia in the proband and healthy controls. (DOCX 53 kb) [file 12883_2018_1199_MOESM2_ESM.docx]
