# Supplementary material for: Identification of novel compound heterozygous SPG7 mutations-related hereditary spastic paraplegia in a Chinese family: a case report
Source: BMC Neurol. 2018 Nov 29;18:196. doi: 10.1186/s12883-018-1199-9 (PMC6263041; doi:10.1186/s12883-018-1199-9)
Supplement: Supplementary file 4 — Table S3. Family verification results. It is the first generation verification results of the variants mentioned in the manuscript in this family including three patients and the asymptomatic subjects. (DOCX 23 kb) [file 12883_2018_1199_MOESM4_ESM.docx]

**Table S3** Family verification results.

|  | | | | | | | | | | | | | |
| --- | --- | --- | --- | --- | --- | --- | --- | --- | --- | --- | --- | --- | --- |
| The results of the first-generation sequencing verification are as follows: | | | | | | | | | | | | | |
|  | Gene | Site | Reference gene | II-1  (The index case) | II-2 | II-3 | II-4 | III-1 | III-2 | III-3 | III-4 | III-5 |  |
|  | *AFG3 L2* | c.C316T | CC | CT | CC | CT | CC | CT | CC | CC | CC | CC |  |
|  | *PPP2 R2B* | rs56261308 2 | GG | GA | GA | GA | GA | GG | GG | GA | GG | GG |  |
|  | *SLC1 A3* | rs11758869 7 | AA | AG | AA | AG | AA | AA | AA | AA | AA | AA |  |
|  | *SPG7* | c.C2062T | CC | CT | CC | CT | CT | CC | CC | CC | CC | CC |  |
|  | *SPG7* | rs12960 | GG | GA | GG | GA | GA | GG | GG | GG | GG | GG |  |
|  | *SPG7* | c.1150_1150-1  insCTAC | CC | C/GCT AC | C/GCT AC | C/GC  TAC | C/GCT  AC | C/GC  TAC | C/GC  TAC | CC | C/GC  TAC | C/GC  TAC |  |
